# Effects of eslicarbazepine acetate on acute and chronic latrunculin A-induced seizures and extracellular amino acid levels in the mouse hippocampus

**DOI:** 10.1186/s12868-014-0134-2

**Published:** 2014-12-20

**Authors:** Germán Sierra-Paredes, Ana I Loureiro, Lyndon C Wright, Germán Sierra-Marcuño, Patrício Soares-da-Silva

**Affiliations:** Department of Biochemistry and Molecular Biology, School of Medicine, University of Santiago de Compostela, Santiago de Compostela, Spain; Department Research & Development, BIAL – Portela & Cª – S.A., 4745-457 S. Mamede do Coronado, Portugal; Department Pharmacology & Therapeutics, Faculty of Medicine, University of Porto, Porto, Portugal; MedInUP - Center for Drug Discovery and Innovative Medicines, University of Porto, Porto, Portugal; Department of Research and Development, BIAL, À Av. da Siderurgia Nacional, 4745-457 S. Mamede do Coronado, Portugal

**Keywords:** Anticonvulsant drugs, Eslicarbazepine acetate, Eslicarbazepine, Latrunculin A-induced seizures, Taurine, Glycine, Aspartate, Glutamate

## Abstract

**Background:**

Latrunculin A microperfusion of the hippocampus induces acute epileptic seizures and long-term biochemical changes leading to spontaneous seizures. This study tested the effect of eslicarbazepine acetate (ESL), a novel antiepileptic drug, on latrunculin A-induced acute and chronic seizures, and changes in brain amino acid extracellular levels. Hippocampi of Swiss mice were continuously perfused with a latrunculin A solution (4 μM, 1 μl/min, 7 h/day) with continuous EEG and videotape recording for 3 consecutive days. Microdialysate samples were analyzed by HPLC and fluorescence detection of taurine, glycine, aspartate, glutamate and GABA. Thereafter, mice were continuously video monitored for two months to identify chronic spontaneous seizures or behavioral changes. Control EEG recordings (8 h) were performed in all animals at least once a week for a minimum of one month.

**Results:**

Oral administration of ESL (100 mg/kg), previous to latrunculin A microperfusion, completely prevented acute latrunculin A-induced seizures as well as chronic seizures and all EEG chronic signs of paroxysmal activity. Hippocampal extracellular levels of taurine, glycine and aspartate were significantly increased during latrunculin A microperfusion, while GABA and glutamate levels remained unchanged. ESL reversed the increases in extracellular taurine, glycine and aspartate concentrations to basal levels and significantly reduced glutamate levels. Plasma and brain bioanalysis showed that ESL was completely metabolized within 1 h after administration to mainly eslicarbazepine, its major active metabolite.

**Conclusion:**

ESL treatment prevented acute latrunculin A-induced seizures as well as chronic seizures and all EEG chronic signs of paroxysmal activity, supporting a possible anti-epileptogenic effect of ESL in mice.

## Background

Eslicarbazepine acetate (ESL) is a once-daily anticonvulsant approved in 2009 by the European Medicines Agency (EMA) and in 2013 by the Food and Drug Administration (FDA) as adjunctive therapy in adults with partial-onset seizures (POS). The ESL epilepsy clinical program included an initial proof of concept phase II study [1] and four subsequent phase III studies in patients refractory to conventional antiepileptic drug (AED) therapy [2-5]. Long-term safety and maintenance of therapeutic effect was demonstrated in one-year open-label extensions of these studies [6-8].

Following oral administration, ESL undergoes extensive first pass hydrolysis to its major active metabolite eslicarbazepine (also known as (S)-licarbazepine) [9-13], which represents approximately 95% of circulating active moieties and is believed to be responsible for its antiseizure effects [14-16] most likely through reduction of VGSC availability due to enhancement of slow inactivation of voltage-gated sodium channels and blockade type T calcium channels [17-19].

Latrunculin A is an actin depolymerization agent [20] that has been widely used to study the role of F-actin in anchoring NMDA receptors to synaptic sites [21,22]. Latrunculin A treatment promotes neurotransmitter release in hippocampal synapses [23] and induces multiple action potential firing and increases action potential duration [24]. Latrunculin A hippocampal microperfusion in freely moving rats induces acute epileptic seizures, followed by the occurrence of chronic sporadic spontaneous seizures, in association with changes in extracellular concentrations of excitatory and inhibitory amino acids [25,26]. In the rat, ESL was found to prevent acute seizures induced by latrunculin A hippocampal microperfusion and reduce the increases in glutamate and aspartate induced by latrunculin A [25]. However, rats, in contrast to mice and humans, extensively convert ESL to oxcarbazepine [27].

The aim of the present study was to evaluate the effects of ESL on the development of latrunculin A hippocampal perfusion-induced acute and chronic seizure model in the mouse and to further examine the effects upon hippocampal extracellular levels of taurine, glycine, aspartate, GABA and glutamate, as changes in the extracellular levels of these amino acids play determinant effects in the neuropathophysiology of epileptic seizures [28-39]. Mice were used for the experiments as the metabolism of ESL in this species reflects the situation observed in humans [27,40,41].

## Results

### MES test

**Figure 1:**
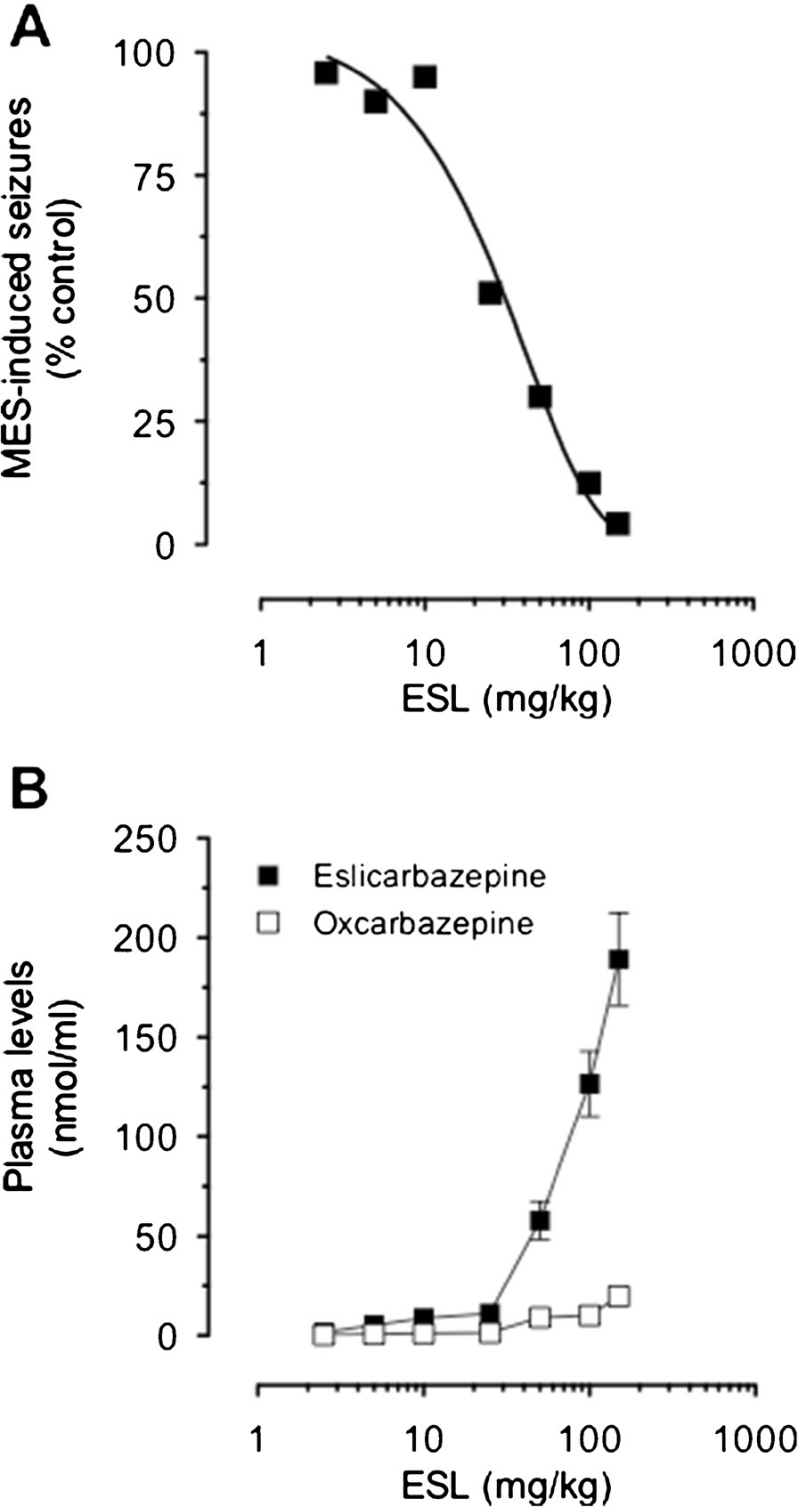
**Antiseizure effects of ESL and exposure to eslicarbazepine.** **A)** Anticonvulsant dose-response curves for ESL against tonic convulsions in the mouse MES test seizure model. Symbols represent mean values of 12-15 animals per group. **B)** Plasma concentrations of eslicarbazepine and oxcarbazepine after oral administration ESL. Symbols represent mean values of 4 animals per group; vertical lines indicate SEM.

Male NMRI mice (n = 12-15 per group) were administered MES (50 mA, rectangular current: 0.6 ms pulse width, 0.4 s duration, 50 Hz) via corneal electrodes connected to a constant current shock generator (Ugo Basile 7801) and the number of tonic convulsions recorded. ESL (2.5 to 150 mg/kg) decreased in dose dependent manner MES-induced seizures (Figure 1A) with an ED_50_ values of 23.0 ± 1.9 mg/kg. Following oral administration of ESL, the major metabolite in plasma obtained from a statelite group of mice was eslicarbazepine, followed by relatively low amounts of OXC (Figure 1B); no trace amounts of ESL and R-licarbazepine were detected. The ESL dose of 100 mg/kg administered 60 min before the test resulted in 90% protection against MES-induced seizures. The concentration of eslicarbazepine in plasma associated with the 90% protection against MES-induced seizures was 126.6 ± 16.4 nmol/ml. Because this dose of ESL was found not to affect the behavioural performance in the rotarod test (data not shown), subsquent experiments (see below) were performed with the 100 mg/kg ESL dose.

### EEG and behavioral observations

No seizures were observed during the first and second day of latrunculin A hippocampal microperfusion. However, all animals (n = 8 per group) studied showed a variable number of seizures (4.6 ± 2.8) on the third day of latrunculin A hippocampal microperfusion. ESL (100 mg/kg), administered 5–10 min before starting latrunculin A hippocampal perfusion, completely prevented latrunculin A induced seizures in all mice studied.

**Figure 2:**
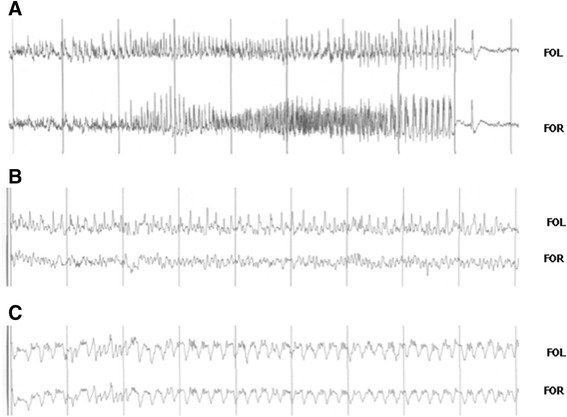
**Chronic effect of latrunculin A hippocampal microperfusion of in freely moving mice. A)** EEG recording of a spontaneous seizure (FOR, fronto-occipital right; FOL, fronto-occipital left). **B)** Contralateral spikes recorded two weeks after latrunculin A hippocampal microperfusion. **C)** Slow wave activity recorded during the third week after latrunculin A hippocampal microperfusion.

During the first week after latrunculin A treatment no behavioral signs or paroxysmal EEG activity was observed, though sporadic periods of slow waves were observed. From the second week onwards, various EEG signals of paroxysmal activity (electrographic seizures, focal and bilateral spike and wave discharges and long periods of slow wave activity) were recorded in all animals studied. Three different types of paroxysmal activity were observed in the same animal (Figure 2): 1) short bilateral electrographic seizure appearing sporadically during the second week after latrunculin A hippocampal microperfusion (typically 2 or 3 events during the 8 hours of control recording, but without accompaning convulsions or absence-like behavior); 2) focal spike discharges observed the third week after latrunculin A treatment, not related to observable behavioral signs; 3) slow waves/polyspikes observed during the third and fourth day of control (consisting of more than 30% of the 8-hours EEG recording, and occurring more frequently during sleep).

**Figure 3:**
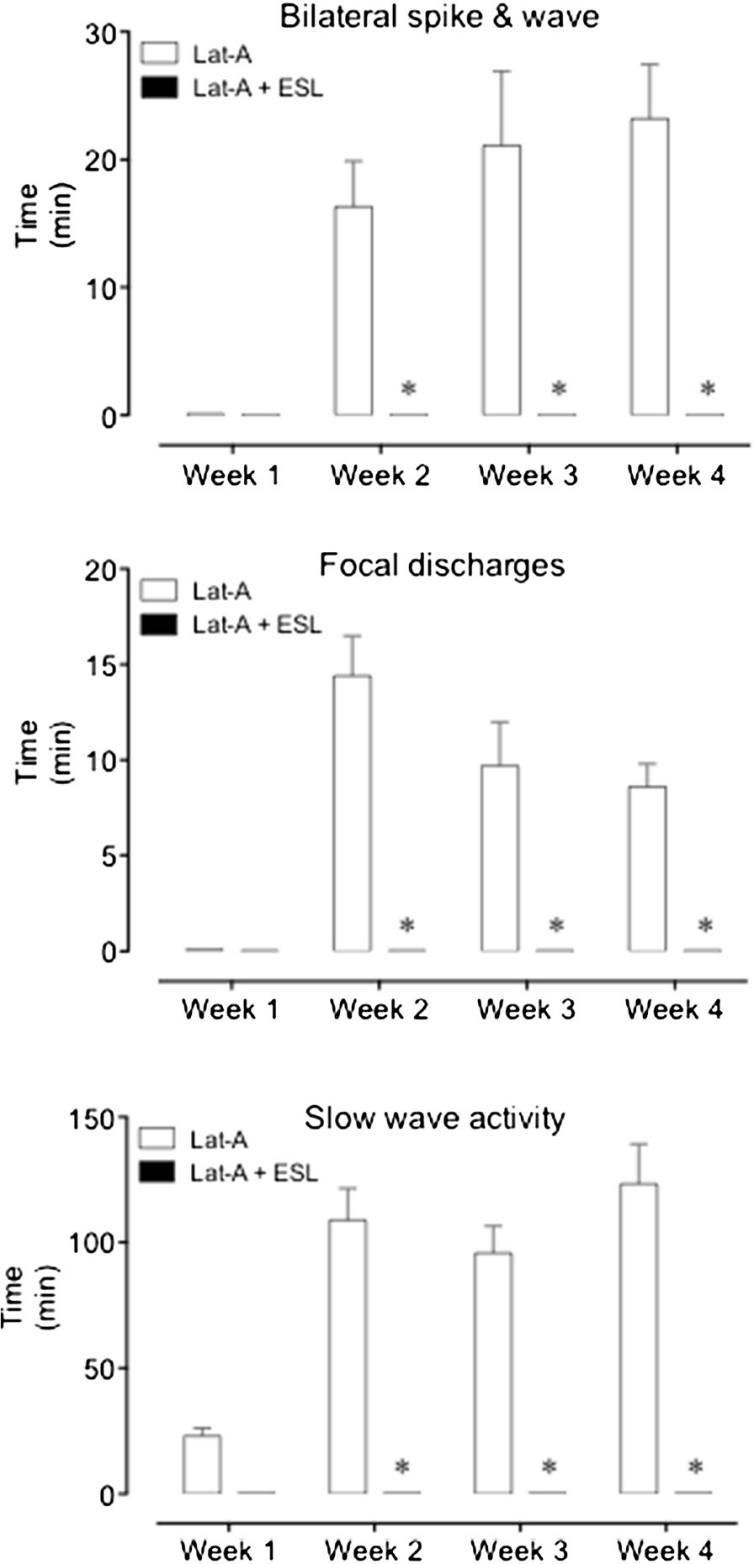
**Average duration of the weekly EEG epileptiform activity (bilateral spike and wave discharges, focal discharges and slow wave activity) recorded during the four weeks following latrunculin A hippocampal microperfusion in vehicle-treated and ESL-treated mice.** Columns represent mean values of 8 animals per group; vertical lines indicate SEM. Significantly different from corresponding values in latrunculin A-treated mice using the two-way ANOVA followed by Holm-Sidak’s multiple comparisons test (*P < 0.001).

EEG epileptiform activity during the four weeks following latrunculin A hippocampal microperfusion was summarized weekly according to time spent (duration) in bilateral spike and wave discharges, focal discharges or slow wave activity (Figure 3). Among the mice studied, electrographic seizures and discharges occurred far more frequently than corresponding behavioral seizures could be detected. One tonic-clonic seizure was observed in two of the animals studied during behavioral observation, but no EEG records were obtained. Bilateral spike and wave discharges, focal discharges or slow wave activity resulting from latrunculin A hippocampal microperfusion were significantly lower in ESL-treated mice than in vehicle-treated mice (P < 0.001, using the by two-way ANOVA followed by Holm-Sidak’s multiple comparisons test; Figure 3).

**Figure 4:**
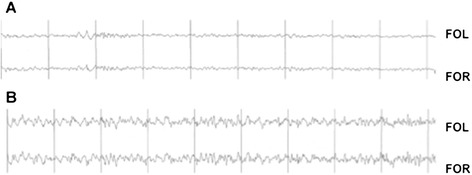
**EEG recordings during wake (A) or sleep (B) in a mouse treated with ESL (100 mg/kg) prior to latrunculin A microperfusion.**

In the animals treated with ESL, no paroxysmal EEG activity was observed during wakefulness and sleep in the month following latrunculin A microperfusion (Figure 4). No observable seizures or behavioural abnormalities were observed during the continuous video recordings of ESL-treated animals.

The inter-rater variability in the assessment of videotaped seizures and behavioral changes was approximately 2%.

### Extracellular amino acid levels

Hippocampal extracellular aspartate (Asp), glutamate (Glu), glycine (Gly), GABA and taurine (Tau) were obtained with a 4-mm microdialysis membrane at a flow rate of 2 μl/min and were corrected for probe recovery. The recovery of Asp, Glu, Gly, GABA and Tau across the dialysis membrane was determined by perfusing modified Ringer solution at a rate of 2 μl/min through standard amino acid solutions ranging from 0.5 to 100 mM. Samples were collected every 15 min and determined as described. Relative probe recovery values (% mean ± SEM) were 23.3 ± 2.3 for Asp, 24.0 ± 3.0 for Glu, 33.2 ± 3.9 for Gly, 21.1 ± 2.3 for GABA and 14.6 ± 5.7 for Tau.

**Figure 5:**
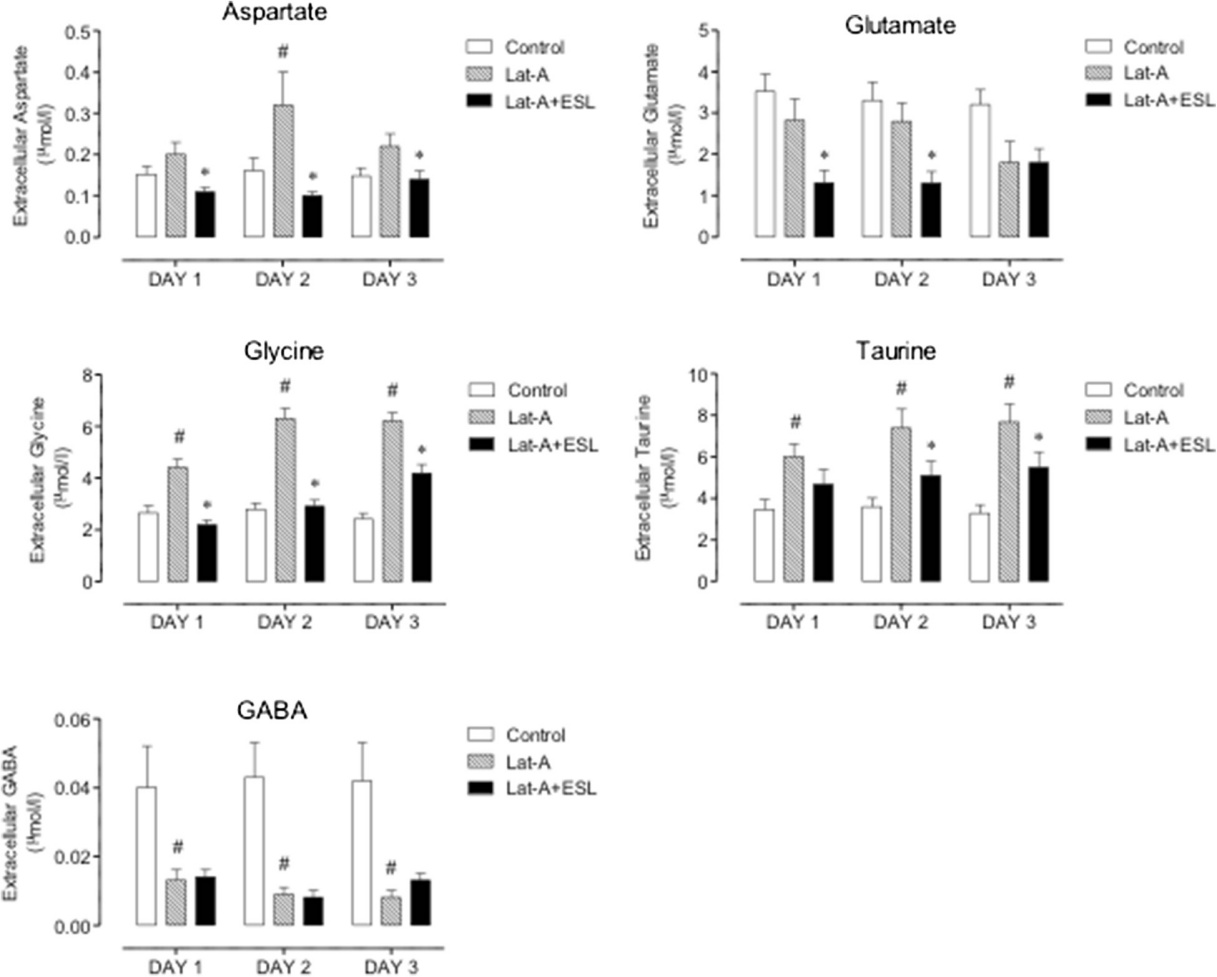
**Extracellular aminoacid concentrations in control mice, after latrunculin A, and latrunculin A and ESL (100 mg/kg). Columns represent mean values of 8 animals per group; vertical lines indicate SEM.** Significantly different from corresponding values in control (# P < 0.05) or latrunculin A-treated mice (*P < 0.05) using the two-way ANOVA followed by Holm-Sidak’s multiple comparisons test.

Basal dialysate concentrations (mean ± SEM) were 0.15 ± 0.02 μmol/l for Asp, 3.53 ± 0.42 μmol/l for Glu, 2.66 ± 0.27 μmol/l for Gly, 0.04 ± 0.01 μmol/l for GABA and 3.54 ± 0.44 μmol/l for Tau. As shown in Figure 5, latrunculin A hippocampal microperfusion induced significant (P < 0.001) increases in extracellular concentrations of Gly and Tau on days 1, 2 and 3 of latrunculin A hippocampal microperfusion, as well as a significant (P < 0.05) decrease in GABA levels, when were compared to control animals. Asp concentrations were significnatly (P < 0.01) increased during day 2 of perfusion. Glu levels remained unchanged during the two first days of perfusion, but decreased during the third day (P = 0.0553). The treatment with ESL prevented the latrunculin A-induced increase in Gly, Asp and Tau microdialysate concentrations (Figure 5). Though perfusion with latrunculin A did not affect extracellular concentrations of Glu during the first 2 days of treatment, ESL nevertheless markedly decreased the concentrations of Glu in the microdialysate of latrunculin A perfused mice during days 1 (P = 0.023) and 2 (P = 0.024). On the other hand, the marked decrease in GABA concentrations in the microdialysate of latrunculin A perfused mice was not affected by ESL administration.

Taking into account that seizures develop on the third day of perfusion, the main changes associated with the epileptiform EEG activity were decreases in GABA and Glu concentrations as well as significant increases in Gly and Tau.

### ESL and metabolite pharmacokinetics

**Figure 6:**
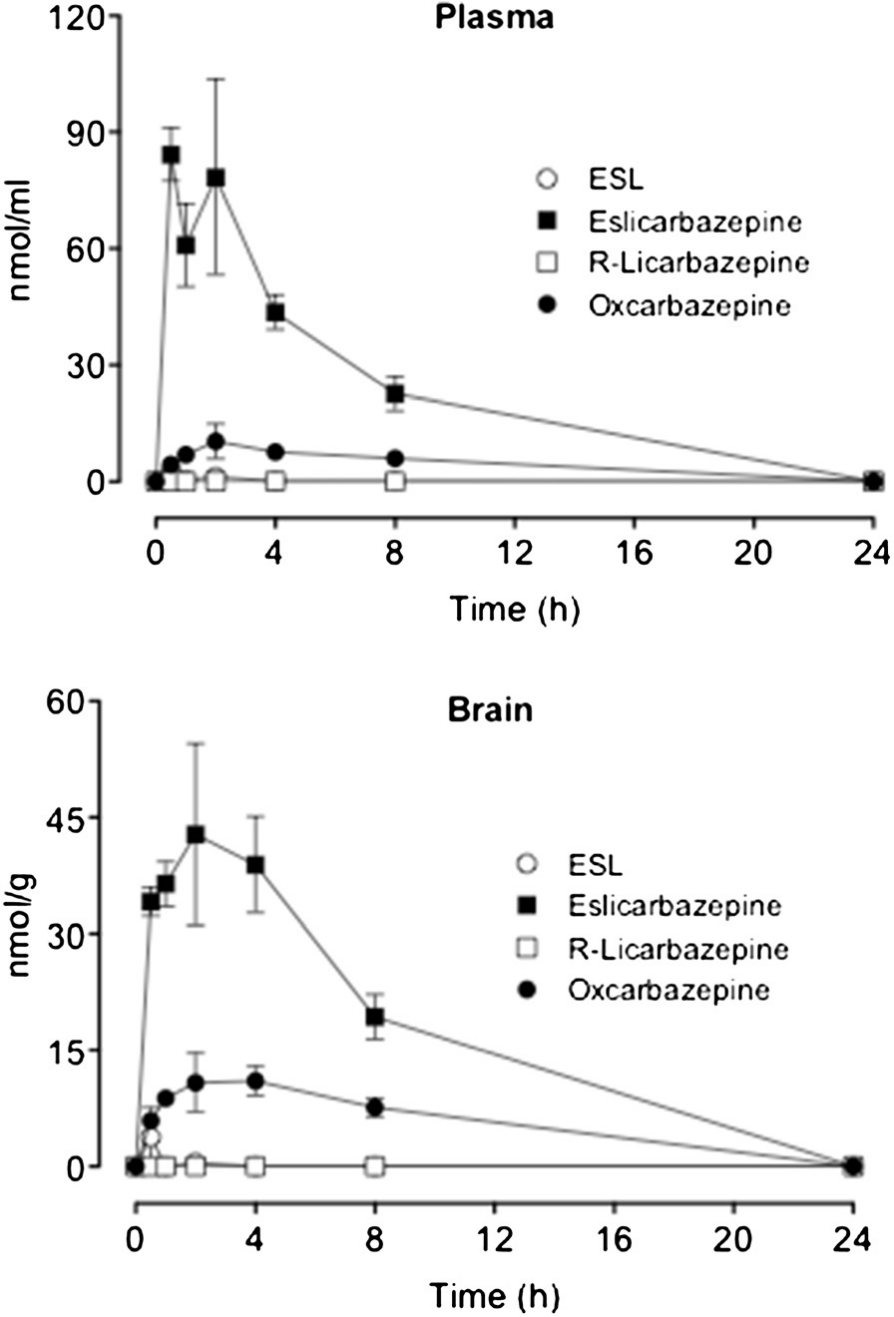
**Plasma and brain concentrations of eslicarbazepine acetate (ESL), eslicarbazepine, (R)-licarbazepine and oxcarbazepine (OXC) after oral administration ESL (100 mg/kg).** Columns represent mean values of 4 animals per group; vertical lines indicate SEM.

Following oral administration of ESL (100 mg/kg), the major metabolite in brain and plasma was eslicarbazepine. Relatively low amounts of OXC were quantified in brain and plasma. Only trace amounts of ESL were detected in brain and no R-licarbazepine was detected. Eslicarbazepine reached maximal concentrations (t_max_) between 0.5 to 4 h following administration and at 8 h eslicarbazepine was in the elimination phase, reaching undetectable levels 24 h following administration (Figure 6). Previously, ESL was tested in mice at 20 mg/kg [40]. In the present study we tested a dose of ESL 5 fold that previously tested. The plasma and brain profile of ESL and metabolites is similar in the two studies.

## Discussion

The present study determined the effects of ESL on epileptogenesis in the mouse latrunculin A model. Moreover, brain amino acid concentrations in the hippocampal microdialysates and ESL and metabolites in brain and plasma pharmacokinetic data were obtained for the pretreatment times and administration routes used in the present study. As previously described for mice [40,41], ESL was extensively converted to eslicarbazepine, as has been observed in humans [9-13]. In contrast to mice and humans, rats extensively convert ESL to oxcarbazepine [27]. ESL significantly prevented latrunculin A-induced seizures and significantly prevented chronic paroxysmal EEG activity in hippocampal latrunculin A-perfused mice. These data indicate that ESL inhibits seizure initiation and can protect against latrunculin A-induced epileptogenesis. This dose of ESL (100 mg/kg), which significantly increased the seizure threshold in latrunculin A-perfused mice, was similar to the dose that prevented the development of seizures in the maximal electroshock test, and on partial-onset seizures in corneal and amygdala kindling models [42].

These results suggest that ESL and eslicarbazepine (the major metabolite after ESL administration) have the potential to prevent latrunculin A-induced epileptogenesis. The observed effects may reflect an anticonvulsant effect on partial onset seizures. However, disease-modifying effects may also be involved. The mechanisms of latrunculin A convulsant action are not completely understood, but the increase in the extracellular concentrations of excitatory amino acids acting on NMDA glutamate receptors, such as Glu, Asp and Gly, seem to be involved in latrunculin A-induced changes in seizure threshold [43,44]. It has been suggested that changes in extracellular hippocampal Glu, Asp and GABA levels are not involved in seizure onset, but may play a role in seizure maintenance and/or spread in some animal models of epilepsy [45]. In fact, high concentrations of Glu and Gly are convulsant in rats treated with intrahippocampal microperfusion of latrunculin A. This effect can be reversed by NMDA antagonists [46], indicating that increased Glu and Gly extracellular levels may be involved in seizure initiation in latrunculin A-induced seizures. The present study in mice shows that changes in extracellular concentrations of brain amino acids during latrunculin A hippocampal microperfusion are not identical to that observed in rats [25]. This is particularly relevant for Glu, where no increase in the extracellular concentrations were observed in the mouse. Another interesting difference between the rat and the mouse relates to the marked decrease in the extracellular concentrations of GABA in latrunculin A-treated mice, which did not occur in the rat [25]. The differences between species in what relates to changes in the extracellular concentrations of Glu and GABA during latrunculin A hippocampal microperfusion, for which no particular reason is apparent, do not appear to play a relevant role with respect to induction of seizures. However, it can be hypothesized that the decrease in the extracellular concentrations of GABA during latrunculin A hippocampal microperfusion may play a role in seizure initiation, maintenance and arrest [44,47,48]. On the other hand, the latrunculin A-induced increase in the extracellular concentrations of Asp and Gly appear to be common features in rats and mice. The observation that ESL treatment prevented latrunculin A-induced increases in the extracellular concentrations of Asp and Gly, without changes in GABA levels, and the development of chronic seizures indicates that the drug may not merely suppress seizure activity but may also inhibit the generation of a constant hyperexcitable epileptic network, resulting in decreases in the extracellular levels of excitatory brain amino acids.

The delayed appearance of bilateral spike and wave discharges, focal discharges or slow wave activity may result from changes in receptor signalling or changes in synaptic connectivity of surviving cells. Multiple changes occur in the days and weeks after convulsant application. Long-term changes in receptor expression have been documented at both excitatory [49,50] and inhibitory synapses [51]. Reactive changes have also been demonstrated in the expression, phosphorylation and distribution of membrane channels that regulate cellular excitability [52-54].

ESL is only hydrolysed to eslicarbazepine in humans and mice [27], whereas oxcarbazepine serves as a prodrug of both 10-hydroxy metabolites, i.e. eslicarbazepine and (R)-licarbazepine, which appear in the plasma and urine in a 4:1 ratio [55,56]. In this context, it is of specific interest to compare the brain availability of both enantiomers. Whereas eslicarbazepine was the major metabolite in brain and plasma, (R)-licarbazepine represented less than 1% of the total amount of ESL metabolites in brain. These data fit well with the finding that eslicarbazepine was more potent in the MES test as compared to the (R)-enantiomer [42]. Though (R)-licarbazepine is endowed with effects as an active voltage-gated sodium channel (VGSC) blocker [18], this may not suffice to guarantee efficacy of this entity as an anticonvulsant. Considerable differences in the pharmacodynamic properties between eslicarbazepine and (R)-licarbazepine have been observed. The affinity of (R)-licarbazepine for VGSCs in the resting state versus the inactive was found to be 4-fold that for eslicarbazepine [18], which may favor a better efficacy of the later over the former. Another remarkable difference between eslicarbazepine and (R)-licarbazepine is concerned with the potency of (R)-licarbazepine as a blocker of voltage-gated potassium-channels, namely K_V_7.2 currents, whereas eslicarbazepine is devoid of effect [57]. In fact, activation of voltage-gated potassium-channels (K_V_7/M) during the initial stages of an action potential discharge suppresses later action potentials and inhibition of channel activity strongly enhances repetitive firing [58]. K_V_7.2 knock-out mice have a reduced electroconvulsive threshold and increased sensitivity to convulsing agents [59]. Finally, eslicarbazepine, but not (R)-licarbazepine, was effective in retarding kindling development during bilateral corneal stimulation in mice [42]. This potential for eslicarbazepine to elicit antiepileptogenic effects, in contrast to (R)-Licarbazepine, may relate to the ability of eslicarbazepine to effectively inhibit high and low affinity Ca_v_3.2 inward currents [17].

Numerous animal models of epileptogenesis have been developed and used in research studies designed to increase our understanding on the molecular abnormalities underlying neuronal hyperexcitability and spontaneous seizures [60]. The latrunculin A-induced acute and chronic seizure model is an alternative model for studying epileptogenesis, as seizures are not the consequence of a previous s*status epilepticus*, but rather depend on the long-term changes in neuronal excitability leading to the onset of sporadic spontaneous seizures [25,26]. However, to understand better the nature of the putative antiepileptogenic effect of ESL there is a need to evaluate whether ESL administered after latrunculin A administration also prevents the occurrence of chronic seizures. The effects of ESL and its 10-hydroxy metabolite eslicarbazepine in this model fit well with the outcome of the clinical experience with ESL in epilepsy also considering data from the initial proof‐of‐concept phase II study [1] as well as three subsequent phase III studies in patients refractory to conventional AED therapy [2-4]. Long-term safety and maintenance of therapeutic effect was demonstrated in one-year open-label extensions of these studies [6,7].

## Conclusion

The data presented here demonstrate anticonvulsant effects of ESL on the development of latrunculin A-induced seizures and paroxysmal EEG activity. ESL may also exhibit disease-modifying effects beyond suppressing just seizure activity, possibly by altering the development of a hyperexcitable network, as evidenced by the suppressed changes in glycine, taurine, glutamate and aspartate concentrations associated with an antiepileptogenic effect.

## Methods

### Animals

Adult NMRI (for MES test) or Swiss male mice, obtained from Harlan-Interfauna or the animal facility of the University of Santiago de Compostela, Barcelona, Spain, with a body weight range of 35–40 g were habituated for at least 5 days after delivery in macrolon cages (25 × 19 × 13 cm; 10 animals per cage) on wood litter with free access to food and water. Before starting the experiments, mice were housed in individual cages under controlled environmental conditions (ambient temperature 21 ± 2°C, humidity 50-60%, 12:12 light/dark cycle) with free access to food and water, except during testing. All experiments were performed in laboratories with controlled environmental conditions and at the same time in the morning to avoid circadian variations. All efforts were made to minimize animal suffering, and chronic animal protocols were designed to reduce the number of animals used. The experiments (GSP2010/003) were authorised by the Ethics Committee of the University of Santiago de Compostela (Spain) and Direção Geral de Alimentação e Veterinária (Portugal) and carried out according to procedures for handling and caring of animals followed the Directive 2010/63/EU of the European Parliament on the protection of animals used for scientific purposes and the Spanish and Portuguese law on animal welfare. All efforts were made to minimize animal suffering, and chronic animal protocols were designed to reduce the number of animals used.

### Maximal electroshock test

Male NMRI mice (n = 12-15 per group) were administered MES (50 mA, rectangular current: 0.6 ms pulse width, 0.4 s duration, 50 Hz) via corneal electrodes connected to a constant current shock generator (Ugo Basile 7801) and the number of tonic convulsions recorded [61]. Vehicle (0.2% hydroxypropylmethylcellulose in distilled water) and ESL were administered orally, at a volume of 10 ml/kg body weight, 60 minutes before the test. Blood samples were taken from satellite groups of mice 60 minutes after ESL administration.

### EEG and microdialysis

Mice (n = 8 per group) were anaesthetized with pentobarbital and placed in a stereotaxic instrument (D. Kopf, Tujunga, CA, USA). Under aseptic conditions, 2 stainless steel microscrews serving as electrodes for EEG recording were positioned in the skull above the frontal and occipital areas of each hemisphere; the reference electrode was anchored in the mid-line. The intracerebral guide for the microdialysis probe was implanted vertically into the ventral hippocampus. Stereotaxis coordinates were 1.75 mm posterior, 2.3 mm lateral and 1.7 mm ventral for the tip of the cannula relative to bregma and dural surface. Wires from the microscrews were soldered to a miniature plug (Cannon MD1-9SL1, ITT Cannon, Santa Ana, USA) and fixed firmly to the skull with dental cement. The experiments were carried out on conscious, freely moving mice 10 days after surgery. Bipolar cortical EEGs were recorded using digital EEG system with continuous video monitoring. Videos were analyzed by two observers blinded to the data.

We used a CMA/120 system for freely moving animals with CMA/7 microdialysis probes. The probe was connected via polyethylene tubing to a syringe selector (CMA/111), and to 1 ml syringes mounted on a microinjection pump (CMA/100). Before starting each experiment, the probe was perfused with ethanol and distilled water. After checking the integrity of the probe under a light microscope, vehicle solution was perfused for 10 min and in vitro recovery was tested routinely before each experiment prior to placement in the mouse hippocampus via a chronically implanted intracerebral guide.

### Treatments

The experiments were performed on freely-moving mice 10 days after surgery. After the fourth day mice were placed daily for three hours in the experimental unit for habituation. Bipolar cortical EEGs were recorded using a digital EEG system (MaxG, Zamora, Spain). In control experiments, vehicle consisting of modified Ringer’s solution containing 1:18750 ethanol was perfused at a constant flow rate of 1 μl/min for 7 h (from 09:00 am to 16:00 pm) over three consecutive days (days 11 to 13 after surgery). Latrunclin A-treated mice were perfused with latrunculin A (4 μg/ml) following the same protocol; 7 h (from 09:00 am to 16:00 pm) over three consecutive days (days 11 to 13 after surgery). The dose of latrunculin A and the place of probe implantation were selected based on the previous experience in freely moving rats, in which latrunculin A hippocampal microperfusion resulted in acute epileptic seizures as well as long term in neuronal excitability leading to the onset of sporadic spontaneous seizures accompanied by changes in extracellular amino acid concentration in the hippocampus [25,26]. Latrunculin A-treated mice received orally via gavage vehicle or ESL (100 mg/kg/day) suspended in water/carboxymethylcellulose 5–10 min before starting each latrunculin A perfusion.

Following treatment, animals were continuously monitored by video over one month, and the video records were reviewed daily for indications of chronic spontaneous seizures or any behavioral changes. Control EEG recordings (8 h; from 09:00 am to 17:00 pm) were performed in all animals at least once per week over a minimum of one month.

### Analysis of amino acids

Microdialysate samples were collected every 30 min over 6 hour period and sample analysis was performed by HPLC with fluorimetric detection using the AccQ-Tag method (Waters) for amino acids, as previously described by Liu et al., [62] and Oreiro-García et al., [63]. In brief, the HPLC system used was a Waters Alliance consisting of a 2695 separation model, a thermostat-controlled column oven, a system interface module, and a 2475 scanning fluorescence detector (all Waters components, Millipore, Milford, MA, USA). A waters Empower 5.0.0. Chromatography Manager was used to control system operation and results management. Fluorescence detection monitored excitation at 250 nm and emission at 395 nm. In an analysis for aspartate (Asp), glutamate (Glu), glycine (Gly), taurine (Tau), 10 μl of standards or sample was buffered to pH 8.8 with 10 μl of AccQ-Fluor borate buffer. In an analysis for GABA, 15 μl of standards or sample was buffered to pH 8.8 with 5 μl of AccQ-Fluor borate buffer. In both, the derivatives were formed with 5 μl of AQC solution. The reaction of AQC with all primary and secondary amines was rapid and excess reagent was hydrolyzed within 1 min. Separation of Asp, Glu, Gly and Tau, was carried out using a 3.5 μm XBridge C18 column (100 mm × 3.0 mm i.d.) from Waters. Eluent A was aqueous acetate phosphate buffer prepared by diluting AccQ-Tag eluent A concentrate with water to a ratio of 1:10, eluent B was HPLC grade acetonitrile and eluent C was water. The injection volume was 5 μl and the separation was obtained at a flow rate of 0.7 ml/min at 30°C with the following gradient program: initial 100% A; 0.5 min, 99% A; 21 min, 95% A; 21.5 min, 80% acetonitrile and 20% water; 28 min 100% A; all linear, except the steps at 0.5 and 28 min which were a step function. For column regeneration, 6.5 min with 80% acetonitrile and 20% water was sufficient to wash out the column and before the next injection, 100% acetate buffer for 10 min was needed to equilibrate the system. Separation of GABA was carried out using a 2.5 μm XBridge C18 column (50 mm × 2.1 mm i.d.) from Waters. Eluent A was aqueous acetate phosphate buffer prepared by diluting AccQ-Tag eluent A concentrate with water to a ratio of 1:10, eluent B was HPLC grade acetonitrile and eluent C was water. The injection volume was 5 μl and the separation was obtained at a flow rate of 0.35 ml/min at 50°C with the following gradient program: initial 99% A; 1 min, 99% A; 7 min, 97% A; 16 min, 89% A; 16.5 min, 80% acetonitrile and 20% water; 25 min 100% A; all linear, except the steps at 0.5 and 28 min which were a step function. For column regeneration, 8.5 min with 80% acetonitrile and 20% water was sufficient to wash out the column and before the next injection, 100% acetate buffer for 10 min was needed to equilibrate the system. The samples were quantified with accuracy and precision over the analytical range of 0.01 to 1.0 μmol/l for Asp and GABA, 0.1 to 10.0 μmol/l for glycine, glutamate and taurine. The limit of quantification was 0.01 μmol/l for aspartate and 0.1 μmol/l for Glu, Gly and Tau.

### Analysis of ESL metabolites

Brain and plasma concentrations of eslicarbazepine acetate, eslicarbazepine and (R)-licarbazepine were determined using a validated enantioselective LC-MS/MS assay, as previously described [64]. In brief, Brain and plasma concentrations of eslicarbazepine acetate, eslicarbazepine and (R)-licarbazepine were determined using a validated enantioselective LC-MS/MS assay, as previously described [64]. Plasma samples (100 μL) were added with 400 μl of internal standard (ISTD) working solution (2000 ng/ml of 10, 11-dihydrocarbamazepine in phosphate buffer pH 5.6) and then processed by solid phase extraction. After thawing and weighing, 0.1 M sodium phosphate buffer (pH 5.6) was added to the brain samples to give a tissue concentration of 0.1 mg/ml. The samples were then homogenized using a Heidolph DIAX 900 mixer and transferred to 10 ml plastic centrifugation tubes. After centrifugation at 10,000 g for 30 min at 4°C, 0.5 ml of supernatants were added to 0.5 ml ISTD working solution and then processed by solid phase extraction. The samples were placed on an automatic liquid handler (ASPEC-XL4, Gilson) for solid phase extraction. Solid phase extraction cartridges (Oasis, HLB, 30 mg, 1 ml Waters) were conditioned with 1 ml of acetonitrile and then washed twice with 1 ml of water. Specimen (400 μl) were loaded onto the cartridges and the cartridges washed twice with 1 ml of water. After the second wash the cartridges were flushed with an air push of 10 ml at 6 ml/min. The cartridges were eluted twice with 200 μl of methanol with an air push of 2 ml at 6 ml/min. The eluted sample was evaporated until dryness and resuspended into 200 μl of hexane:2-propanol (90:10, v:v). The samples were injected (20 μl) into a LC-MS/MS. The analysis of samples extracts was performed using LC-MS/MS (Quattro Ultima, Waters) with positive ion detection. Separation was performed on a ChiralCel 0.46 cm × 15 cm OD-H column (Chiral technologies, Europe). The mobile phase consisted of an isocratic mixture of hexano:ethanol (80:20, v:v; flow rate of 1 ml/min), and Ethanol with 5 mM ammonium acetate (added post-column using a flow rate of 0.15 ml/min). A volume of 20 μL was injected and the column temperature kept at 50°C for the run time of 7 min. Electrospray ionisation was used for all mass spectrometer methods with a cone voltage of 40 V and capillary current of 3.8 kV. The multiple reaction monitoring pair was m/z 239.1 → 194, collision 40 eV for ISTD; 253.1 → 208, collision 25 eV for oxcarbazepine; 255.1 → 194, 237, collision 30 eV for eslicarbazepine and (R)-licarbazepine; 297.1 → 194, 237, collision 30 eV for eslicarbazepine acetate. The autosampler cooler was maintained at 10°C. The samples were quantified with accuracy and precision over the analytical range of 0.2 to 8.0 nmol/ml for plasma and 2.0 nmol/g to 80 nmol/g for brain. The limit of quantification was 0.2 nmol/ml and 2.0 nmol/g, for plasma and brain respectively.

### Chemicals

Eslicarbazepine acetate [(−)-(S)-10-acetoxy-10,11-dihydro-5H-dibenzo/b,f/azepine-5-carboxamide], eslicarbazepine [(+)-(S)-10,11-dihydro-10-hydroxy-5H-dibenzo/b,f/azepine-5-carboxamide], (R)-licarbazepine [(−)-(R)-10,11-dihydro-10-hydroxy-5H-dibenzo/b,f/azepine-5-carboxamide], 10,11-dihydrocarbamazepine (used as internal standard), and oxcarbazepine, were all synthesized in the Laboratory of Chemistry, BIAL – Portela & Cª, S.A., with purities >99.5%. Latrunculin A was obtained from Molecular Probes (Eugene, Oregon, USA).

### Statistical analysis

Results are presented as mean ± SEM. EEG records were analysed using the Medlog 9200 software, version 7.2. Spike and wave discharges duration, seizure duration, and number of seizures were evaluated. Statistical significance of the difference in number of seizures, seizure duration, and seizure onset times was determined by two-way ANOVA followed by Holm-Sidak’s multiple comparisons test. Significant differences in amino acid levels were determined by two-way ANOVA for repeated measures followed by Holm-Sidak’s multiple comparisons test. P-values less than 0.05 were regarded as statistically significant.

## Abbreviations

AED: Antiepileptic drug
Asp: Aspartate
EEG: Electroencephalohgram
ESL: Eslicarbazepine acetate
GABA: Gamma-aminobutyric acid
Glu: Glutamate
Gly: Glycine
HPLC: High pressure liquid chromatography
POS: Partial-onset seizures
Tau: Taurine
